# Phytochemical Composition and Antinociceptive Activity of *Bauhinia glauca* subsp. *hupehana* in Rats

**DOI:** 10.1371/journal.pone.0117801

**Published:** 2015-02-06

**Authors:** Jinlong Xu, Qizhi Zhao, Lei Wei, Yu Yang, Rui Xu, Nengjiang Yu, Yimin Zhao

**Affiliations:** Department of Natural Product Chemistry, Beijing Institute of Pharmacology and Toxicology, Beijing, China; University of Copenhagen, DENMARK

## Abstract

In traditional medicine, *Bauhinia glauca* subsp. *hupehana* has long been used as an analgesic agent in China. The aim of this study was to evaluate the antinociceptive activity of the ethanol extract of the aerial parts of *B. glauca* subsp. *hupehana* (BHE) in rats and its chemical fingerprint. The antinociceptive activity of BHE was assessed in mice using chemically and heat–induced pain models, such as the acetic acid–induced writhing, hot plate, tail–flick and glutamate tests. Naltrexone hydrochloride, a non–selective opioid receptor antagonist, was utilized to determine the involvement of the opioid system. In addition to this, the involvements of the cGMP and ATP–sensitive K^+^ channel pathways were also detected using methylene blue and glibenclamide. The oral administration of BHE (at doses of 50, 100 and 200 mg/kg) produced significant and dose–related inhibitions in both the chemically and heat–induced pain models. Interestingly, in the abdominal constriction test, when the dose of BHE was increased to 800 mg/kg (p.o., n = 10), the inhibition rate was 100%. The antinociceptive mechanism may involve the cGMP pathway and ATP sensitive K^+^ channel pathway. The central antinociceptive effect was not antagonized by naltrexone. One phenolic acid, one lignin and five flavonoids were isolated from BHE. The antinociceptive activity of BHE was most likely due to the presence of the flavonoids. The acute toxicity results showed that BHE was safe at a high dose (2 g/kg, p.o.). The current investigation demonstrates that *B. glauca* subsp. *hupehana* is a potential candidate for the development of novel, non–opioid, analgesic *phytomedicines*.

## Introduction

In the past decades, many studies have been conducted to investigate analgesic effects. Despite the progress that has been made in the development of pain therapies, it is still necessary to discover new analgesic agents with confirmed efficacy that are devoid of side effects for the treatment of various painful conditions, especially chronic painful diseases [1]. In comparison with the current therapeutic regimes available for the management of pain, both peripheral and centrally acting, natural products that are used to treat pain have an advantage due to little adverse effects [2]. In the early days, some clinically useful analgesic agents were discovered from plants, such as aspirin and morphine. To explore new effective and safe analgesics for the management of different painful conditions, *Bauhinia glauca* subsp. *hupehana* has been studied in various pain models according to its traditional use.

*B. glauca* subsp. *hupehana*, a climbing plant, is widely grown in the south of China and other Asian countries. It is a member of the genus *Bauhinia*, belonging to the family Leguminosae. The genus *Bauhinia* consists of approximately 300 species, which are commonly known as cow’s paw or cow’s hoof because of the shape of their leaves. Their leaves and stem–bark have been used frequently as a remedy for different types of pathologies, particularly diabetes, infections, pain and inflammatory processes [3]. In folk medicine, the leaves, stems and roots of *B. glauca* subsp. *hupehana* were used in the treatment of inflammatory and painful conditions, such as backache, testicular pain, hemostasis swelling pain and rheumatic arthritis [4]. Although *B. glauca* subsp. *hupehana* is a particularly useful pain–relief herb in the traditional system of medicine, there have been few reports on the antinociceptive activity of this plant and its mechanisms of analgesic activity.

Based on the above investigation, we studied the analgesic activity of *B. glauca* subsp. *hupehana* and found that the ethanolic extract of B. *glauca* subsp. *hupehana* (BHE) had powerful antinociceptive activity in preliminary experiments. In this paper, we further examined the effects of BHE on nociception models in mice that were induced by both chemical and thermal stimuli and provided a scientific basis for the clinical use of *B. glauca* subsp. *hupehana*. To our knowledge, this is the first report on the antinociceptive activity of *B. glauca* subsp. *hupehana*.

## Materials and Methods

### Plant material

The aerial parts of *B. glauca* subsp. *hupehana* were collected from District Shen–nong–jia in Hubei Province, China in October 2012. The collected plant material was identified at the Department of Natural Product Chemistry, Institute of Pharmacology and Toxicology (Beijing, China). A voucher specimen with catalogue number #108 was preserved at the Herbarium of the Department of Natural Product Chemistry.

### The sample preparation

The air–dried aerial parts of the plant (14 kg) were chopped into small pieces and powdered. For metabolite extraction the powdered plant material was treated with aqueous ethanol (85% v/v) under reflux three times for 2 h each time. The aqueous ethanol was filtered through filter paper, and the filtrates were concentrated under a vacuum at a low temperature (50°C) using a rotary evaporator. After the removal of the solvent under reduced pressure, the residue (1.1 kg, 7.86% w/w) was obtained for use in the pharmacological tests.

### Animals

Each experimental group consisted of 10 ICR (Institute of Cancer Research, Vital River Laboratory Animal Technology Co. Ltd., Beijing, China) mice above 6–weeks old (18–22 g, equal numbers of male and female). They were housed at 21 ± 1°C under a 12–h light/12–h dark cycle and had free access to standard pellet diet (Purina chow) and tap water. The animals were deprived of food for 15 h before the experiment, with free access to drinking water. All mice received humane care, and each animal was used only once in the experiment. After each experiment, the mice were sacrificed under anesthesia with isoflurane and decapitated to ameliorate any suffering.

### Ethics statement

Specific permissions were not required for the described field sampling studies or for the collection of plant materials. For any locations/activities, no specific permissions were required. All locations where the plants were collected were not privately owned or protected in any way, and the field studies did not involve endangered or protected species.

The experimental protocols were approved by the Institutional Animal Care and Use Committee of the Academy of Military Medical Sciences (Protocol number IACUC of AMMS–2013012, Date: 20130906) and complied with the recommendations of the International Association for the Study of Pain [5]. All efforts were made to minimize suffering.

### Drugs and chemicals

Aspirin (Qingdao Yellow Sea Pharmaceutical Co., Ltd., China), acetic acid (Sinopharm Chemical Reagent Co., Ltd, China), morphine hydrochloride, naltrexone hydrochloride (Chengdu Pharmaceutical Factory, Chengdu, China), L–glutamic acid (GL Biochem, Ltd., China), methylene blue (Sinopharm Chemical Reagent Co., Ltd, China), and glibenclamide (TIPR Pharmaceutical Co., Ltd., China) were all used in this study. For treatment of the control animals, 0.9% NaCl was used.

### High–pressure liquid chromatography analysis

High–pressure liquid chromatography (HPLC) analysis was performed using an Agilent Technologies 1100 Series HPLC Value System with a photodiode array detector and an automatic injector. The column employed was an INLUCK column ODS–AQ; 250 × 10 mm, 5 μm particle size. The solvents that constituted the mobile phase were water (A) and methanol (B). The elution conditions applied were as follows: 0–10 min, 5–40% B; 10–50 min, 40–100% B and 50–60 min, 100–100% B. The mobile phase was returned to the original composition over the course of 60 min, and an additional 5 min were allowed for the column to re–equilibrate before the injection of the next sample. The sample volume was 60 μl at a concentration of 5 mg/ml, the flow rate was 1.5 ml/min, and the temperature was maintained at 25°C during the analysis. Detection was performed simultaneously at 220, 254, 280 and 320 nm. For all experiments, BHE was dissolved in methanol.

### Acute toxicity test

The acute toxicity test for BHE was performed according to the up–and–down method [6]. ICR mice (n = 10) of either sex were administered BHE orally at doses of 500, 1000 and 2000 mg/kg. The dose was increased if the animal survived at the lower dose, and 0.9% NaCl was used for the controls. The animals were observed carefully for any gross effects or mortality during 24 h.

### *In vivo* antinociceptive studies

**Abdominal constriction induced by acetic acid**. After fasting overnight, the mice were randomly divided into six groups, with each group containing ten animals. The analgesic activity of the extract was assessed by the acetic acid–induced writhing method [7]. Group I was treated with 0.9% NaCl (10 ml/kg, p.o.); BHE (50, 100, 200 and 800 mg/kg, p.o.) was administered orally to groups II, III, IV and V, respectively; group VI received aspirin (200 mg/kg, p.o.) as a positive control. After 30 min of administration of the above treatments, each group was treated with 0.7% acetic acid (10 ml/kg, i.p.) to induce the writhes. The nociceptive responses were counted for 12 min, starting 3 min after acetic acid injection. The number of abdominal writhing occurrences was counted as an indication of pain behavior.

**Tail–flick test**. In the tail–flick test [8], an Analgesia Tail–Flick apparatus (UGO BASILE S.R.L., Model: 7360, Italy) consisting of an infrared heater was used. The infrared spectroscopy intensity was adjusted to 50 V. Before treatment, the rear 3.5 cm of each mouse's tail was set at the infrared heater, and the time taken to flick the tail was automatically recorded by the apparatus. The basal latencies of the mice were determined three times in intervals of 30 min. Only mice showing a pre–treatment reaction between 2 and 10 s were selected for the study. After the basal latency assessment, the prescreened animals (n = 10) were arranged into five groups that received 0.9% NaCl (10 ml/kg, p.o.), BHE (50, 100 and 200 mg/kg, p.o.) or morphine (10 mg/kg, s.c., positive control group), respectively. The cut–off time was 30 s in this test in order to minimize tissue injuries. The antinociceptive activity was observed at 0, 30, 60, 90 and 120 min.

**Hot plate test**. The hot plate test was carried out on groups of female mice using a hot plate apparatus (UGO BASILE S.R.L., Model: 7280, Italy) [9]. The temperature of metal hot plate was maintained at 55 ± 0.05°C. The mice were screened using the apparatus, and only mice that showed an initial nociceptive response between 5 and 30 s were selected for the experiment. The latency to the first sign of hind paw licking or jumping to avoid thermal nociception was taken as an index of nociceptive threshold. In this test, pre–treatment latencies were determined three times in intervals of 30 min. The prescreened mice were arranged in five groups (n = 10). Sodium chloride (0.9%, 10 ml/kg) was injected into group I, BHE (50, 100 and 200 mg/kg, p.o.) was administered to groups II, III and IV, respectively, and group V received morphine (10 mg/kg, s.c.), an opioid analgesic, as a standard drug. The response latencies were recorded at 0, 30, 60, 90 and 120 min, and the cut–off time was 60 s in order to minimize skin damage.

**Glutamate–induced nociception**. The method used was similar to that previously reported [10]. Five groups of ten mice each were treated with 0.9% NaCl (10 ml/kg), BHE (50, 100 and 200 mg/kg, p.o.), and aspirin (200 mg/kg, p.o.). Thirty minutes after the administration, 20 μl of glutamate (10 μmol/paw) was injected into the ventral surface of the right hind paw. The mice were observed for 15 min following the glutamate injection. The number of times a mouse licked its injected paw was indicative of nociception.

### Analysis of the possible mechanism of action of BHE

**Involvement of the opioid system**. In order to verify the participation of the opioid system in the antinociceptive effect of BHE, an effective dose of BHE (200 mg/kg) was examined in groups of mice pre–treated with naltrexone hydrochloride (5 mg/kg, s.c.), a non–selective opioid receptor antagonist. Naltrexone hydrochloride was administered 15 min before either BHE or morphine was administered. The hot plate latencies were sequentially measured at 0, 30, 60, 90 and 120 min after dosing with BHE or morphine.

**Involvement of the cyclic guanosine monophosphate (cGMP) pathway**. To detect the possible involvement of cGMP in the antinociceptive action of BHE, the mice were pre–treated with methylene blue (20 mg/kg), a nonspecific inhibitor of NO/guanylylcyclase, intraperitoneally 15 min before the administration of an effective dose of BHE (100 mg/kg). Thirty minutes after the administration of the treatments, each group was treated with 0.7% acetic acid (10 ml/kg, i.p.) to induce the writhes. The nociceptive responses were counted for 12 min, starting 3 min after the acetic acid injection. The number of abdominal writhing occurrences was counted as an indication of pain behavior.

**Involvement of the ATP–sensitive K^+^ channel pathway**. To evaluate the contribution of the K^+^ channel in the antinociceptive effect of BHE, an effective dose of BHE (200 mg/kg) was examined in groups of mice pre–treated with glibenclamide (10 mg/kg, i.p.), an ATP–sensitive K^+^ channel inhibitor. Glibenclamide (i.p.) was administered 15 min before either the control or BHE was administered. Thirty minutes after the administration of the treatments, each group was treated with 0.7% acetic acid (10 ml/kg, i.p.) to induce the writhes. The nociceptive responses were counted for 12 min, starting 3 min after the acetic acid injection. The number of abdominal writhing occurrences was counted as an indication of pain behavior.

### Statistical analysis

The results are presented as the mean ± standard error of the mean (SEM). The statistical analysis was performed using a one–way analysis of variance (ANOVA), followed by the Dunnett's test for multiple comparisons. The results were considered statistically significant when p values were < 0.05.

## Results

### High–pressure liquid chromatography assay

**Fig 1:**
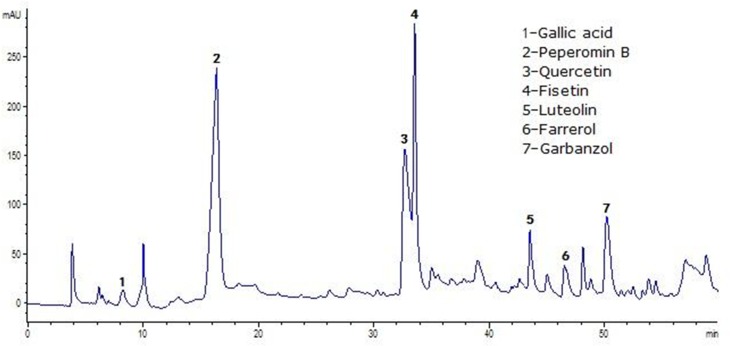
HPLC chromatogram of *Bauhinia glauca* subsp. *hupehana* ethanol extract (BHE) at 254 nm.

The HPLC chromatogram profile for BHE is shown in Fig. 1. The ethanol extract was subjected to silica gel, octadecylsilyl silane (ODS), Sephadex LH–20 chromatography and preparative thin layer chromatography (TLC). For further purification, it was submitted to semi–HPLC. Seven compounds were isolated from BHE. They were identified as gallic acid (percentage composition: 1.0%), peperomin B (19.9%), quercetin (12.3%), fisetin (12.5%), luteolin (3.8%), farrerol (2.3%) and garbanzol (6.8%), according to ^1^H nuclear magnetic resonance (NMR) and mass spectrometry (MS) spectral data. The other compounds have not yet been identified and are currently under investigation in our laboratory. Peperomin B, quercetin, fisetin can be used as markers for BHE because of their high contents. The chemical structures and ^1^H NMR and MS spectral data of compounds 1–7 are presented in the Supporting Information.

### Effect of the acute toxicity test

In the acute toxicity test, BHE was found to be completely safe up to a dose of 2 g/kg (p.o.), and no gross effects or mortality were observed during 24 h for any animal. Therefore, the LD_50_ (lethal dose, 50%) value for BHE in mice was estimated at > 2 g/kg (p.o.). Because the dose used in the acute toxicity test was 20–fold higher than the effective dose used in the other experiments (100 mg/kg, p.o.), it is assumed that the doses given to the mice in this study (50, 100, and 200 mg/kg, p.o.) were safe.

### Effect of abdominal constriction induced by acetic acid

**Fig 2:**
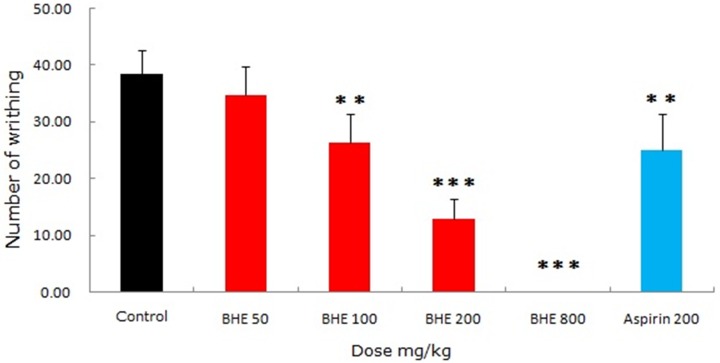
Effects of BHE (50, 100, 200 and 800 mg/kg, p.o.) and aspirin (200 mg/kg, p.o.) on acetic acid-induced writhing in mice. Each column represents the mean ± SEM (n = 10). Asterisks indicate significant difference from control. *p < 0.05, **p < 0.01, ***p < 0.001 (ANOVA followed by Dunnett’s test).

The effects of BHE and aspirin on the writhing response in mice were shown in Fig. 2. The number of writhing occurrences was in the order of 38.44 ± 4.13, 34.63 ± 5.10, 26.30 ± 5.01, 12.78 ± 3.53 and 0 ± 0 for the control (0.9% NaCl) and BHE (50, 100, 200 and 800 mg/kg), respectively. It was found that treatment with BHE significantly decreased the abdominal constriction response based on the mean number of writhes. When the positive drug, aspirin (200 mg/kg, p.o.), was given, a significant reduction in the number of writhes (25.00 ± 6.39) was also demonstrated. The maximum pain relieving effect of BHE was observed at a dose of 800 mg/kg (inhibition rate: 100%), and the analgesic response at a dose of 100 mg/kg was quite similar to that of aspirin. Therefore, BHE exhibited a dose–dependent inhibition of writhing in response to nociception.

### Effect of the tail–flick test

The mean durations within 2 h in the groups of BHE–treated animals (50, 100 and 200 mg/kg) and the control (0.9% NaCl) group are shown in Table 1. The results showed that BHE had a significant effect on the duration the mouse's tail was stimulated by infrared radiation (IR) compared with the control group. Moreover, the positive control group, which was treated with morphine (10 mg/kg), also exhibited a powerful antinociceptive activity. The peak antinociceptive activities of BHE and morphine were observed after 60 and 30 minutes, respectively. The data demonstrated that the antinociceptive activity of BHE was dose–dependent.

**Table 1:** Effects of BHE and morphine stimulated by IR in the tail–flick test.

Groups	Dose	Latency of nociceptive response(s) (mean ± SEM)				
	mg/kg	0 min	30 min	60 min	90 min	120 min
Control	10 ml/kg	5.98 ± 0.58	5.50 ± 0.46	5.29 ± 0.38	5.27 ± 0.50	4.82 ± 0.46
BHE	50	5.77 ± 0.73	6.09 ± 0.91	6.31 ± 1.10 ^a^	5.95 ± 0.77	5.53 ± 0.64
	100	6.05 ± 0.45	6.69 ± 1.08 ^a^	8.94 ± 0.57 ^c^	7.12 ± 1.26 ^b^	6.95 ± 1.37 ^b^
	200	5.95 ± 0.42	8.28 ± 0.67 ^c^	9.93 ± 0.64 ^c^	8.80 ± 0.32 ^c^	7.56 ± 0.45 ^c^
Morphine	10	5.88 ± 0.85	20.33 ± 1.25 ^c^	19.15 ± 0.74 ^c^	15.69 ± 0.63 ^c^	10.67 ± 0.68 ^c^

The data represent the mean ± SEM (n = 10).

^a^ *p* < 0.05

^b^ *p* < 0.01

^c^ *p* < 0.001 (ANOVA, followed by the Dunnett's test).

### Effect of the hot plate test

In the hot plate test, the mean durations of the groups of BHE– (50, 100 and 200 mg/kg), morphine– and control–treated (0.9% NaCl) groups were presented in Table 2. Analgesia was defined by increasing the latency time (s), which was recorded at 0, 30, 60, 90 and 120 min after the different administrations. The results illustrated that BHE (100 and 200 mg/kg) and morphine had powerful antinociceptive effects. The peak protection against thermal pain was achieved at 60 min after BHE administration, and the protection was dose–dependent.

**Table 2:** Effects of BHE and morphine in the hot plate test.

Groups	Dose	Duration on the hot plate(s) (mean ± SEM)				
	mg/kg	0 min	30 min	60 min	90 min	120 min
Control	10 ml/kg	15.63 ± 1.24	14.13 ± 1.21	13.99 ± 0.88	14.21 ± 0.86	14.55 ± 1.19
BHE	50	15.61 ± 1.25	14.94 ± 1.33	18.62 ± 1.42 ^c^	14.79 ± 0.79	14.58 ± 1.15
	100	14.45 ± 0.81	16.85 ± 1.84 ^a^	19.80 ± 1.32 ^c^	15.92 ± 1.24 ^b^	15.54 ± 1.31
	200	14.54 ± 0.86	19.60 ± 0.65 ^c^	23.20 ± 1.25 ^c^	18.07 ± 0.94 ^c^	17.35 ± 1.00 ^c^
Morphine	10	14.52 ± 0.99	33.43 ± 1.67 ^c^	41.68 ± 2.38 ^c^	31.29 ± 1.95 ^c^	30.24 ± 2.49 ^c^

The data represent the mean ± SEM (n = 10).

^a^ *p* < 0.05

^b^ *p* < 0.01

^c^ *p* < 0.001 (ANOVA, followed by the Dunnett's test).

### Effect of glutamate–induced nociception

The result of the glutamate–induced nociception test is shown in Table 3. Compared to the control group (0.9% NaCl), BHE (100 and 200 mg/kg) produced significant inhibition of the glutamate–induced nociception test. Aspirin (200 mg/kg, p.o.) was used as a positive control drug, and it inhibited licking by 53.50% compared to the control group. The data demonstrated that the antinociceptive activity of BHE was dose–dependent.

**Table 3:** Effect of BHE on glutamate–induced nociception.

Treatment (mg/kg)	Licking number (Mean ± SEM)	% Inhibition
Control (10 ml/kg)	167.30 ± 10.67	–
Aspirin (200)	77.80 ± 8.08 ^c^	53.50
BHE (50)	162.60 ± 8.64	–
BHE (100)	123.50 ± 8.40 ^b^	26.18
BHE (200)	59.20 ± 4.96 ^c^	64.61

The data represent the mean ± SEM (n = 10).

^b^ *p* < 0.01

^c^ *p* < 0.001 compared with the control group (ANOVA, followed by the Dunnett's test).

### Involvement of the opioid system

**Fig 3:**
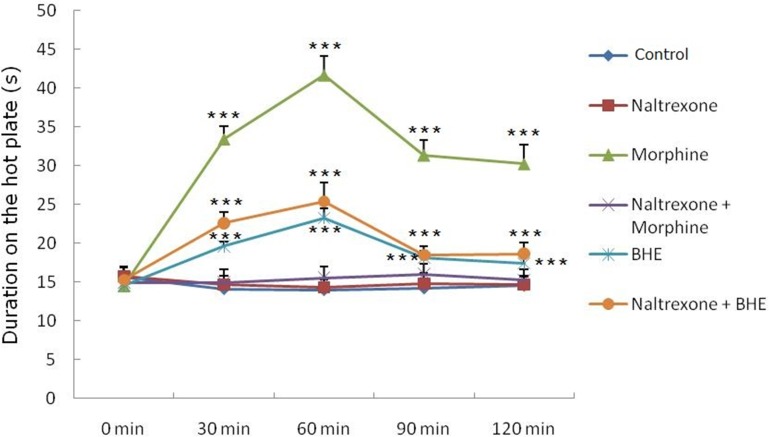
Effects of naltrexone interacted with morphine or BHE thermal-induced antinociception within 2 h in the hot plate test. Naltrexone (5 mg/kg, s.c.) was injected, 15 min prior to the administration of test samples. Control (10 ml/kg) or the BHE (200 mg/kg) was administered orally and morphine (10 mg/kg) subcutaneously. The time in seconds taken to lick the hind paw or jump was recorded. Cut-off time was 60 s. Values are expressed as mean ± SEM (n = 10). ***p < 0.001 vs. control (ANOVA followed by Dunnett's test). The independent t-test was used for comparison between two groups.

The effects of naltrexone interacted with morphine or BHE (200 mg/kg) were presented in Fig. 3. When used alone, the mean of the duration elicited by naltrexone was 14.65 ± 1.13 s. It failed to modify the thermal–induced nociceptive response in a significant manner compared with the control (0.9% NaCl) (14.13 ± 1.21 s). On the other hand, naltrexone was able to significantly reverse the antinociceptive activity of morphine (10 mg/kg, s.c.) but could not abolish the antinociception of BHE in the combination studies.

### Involvement of the cyclic guanosine monophosphate (cGMP) pathway

As shown in Table 4, BHE (100 mg/kg) and methylene blue administration alone significantly inhibited acetic acid–induced abdominal writhing. When used together, methylene blue significantly enhanced BHE–induced (100 mg/kg) antinociception compared to the treatments with BHE and methylene blue alone.

**Table 4:** Effect of BHE on the involvement of the cyclic guanosine monophosphate (cGMP) pathway.

Treatment (mg/kg)	Writhing (Mean ± SEM)	% Inhibition
Control (10 ml/kg)	40.60 ± 4.65	–
Methylene blue (MB) (20)	28.50 ± 5.28*	29.80
BHE (100)	29.40 ± 3.47*	27.59
BHE (100) + MB (20)	14.90 ± 3.78 ^a^	63.30

The data represent the mean ± SEM (n = 10).

MB = Methylene blue.

*p < 0.01 compared with the control group;

^a^ *p* < 0.01 compared with the BHE 100 group (ANOVA, followed by the Dunnett's test).

### Involvement of the ATP–sensitive K^+^ channel pathway

The effects of BHE and glibenclamide on the writhing response in mice are shown in Table 5. It was found that glibenclamide (10 mg/kg) administration alone did not alter abdominal writhing counts compared with the control (0.9% NaCl). Given together, the antinociceptive activity of BHE (200 mg/kg) was markedly decreased by glibenclamide.

**Table 5:** Effect of BHE on the involvement of the ATP–sensitive K^+^ channel pathway.

Treatment (mg/kg)	Writhing (Mean ± SEM)	% Inhibition
Control (10 ml/kg)	41.90 ± 4.07	–
Glibenclamide (G) (10)	38.90 ± 6.82	–
BHE (200)	14.30 ± 3.33*	65.87
BHE (200) + G (10)	32.60 ± 4.12 ^a^	22.20

The data represent the mean ± SEM (n = 10).

G = Glibenclamide.

*p < 0.001 compared with the control group;

^a^ *p* < 0.001 compared with the BHE 200 group (ANOVA, followed by the Dunnett's test).

## Discussion

Investigating plant–derived products is a vital way to discover effective and less toxic drugs. Today, many valuable drugs that are widely used in clinics, such as morphine, taxol and quinidine, were identified through the study of indigenous remedies. In this paper, the plant of *B. glauca* subsp. *hupehana* was selected as a research material for two reasons. Firstly, *B. glauca* subsp. *hupehana* has been used as an analgesic agent in China for hundreds of years. The leaves, stems and roots of *B. glauca* subsp. *hupehana* were utilized for treating different painful conditions. Secondly, we found that BHE had a powerful antinociceptive activity on the abdominal constriction induced by acetic acid, with an inhibition rate of 100% at 800 mg/kg (p.o.). Moreover, the effective antinociceptive dose of BHE (100 mg/kg) was relatively low when compared with other plant extracts with analgesic activity [11] [12]. This combined with the result of the acute toxicity test (BHE at the high dose of 2 g/kg, p.o., was safe), BHE has the potential to be developed as a new therapeutic reagent for pain, and it is worth further study. In addition to this, the interest in plants from the genus *Bauhinia* has increased considerably throughout the world in recent years because experimental studies have confirmed their reported therapeutic properties. The biological properties of different *Bauhinia* spp. and their pure metabolites have been investigated in numerous experimental *in vivo* and *in vitro* models [3].

In the pharmacological experiments, BHE demonstrated remarkable and dose–dependent antinociceptive activity in various pain models, including acetic acid–induced abdominal constriction, the tail–flick test, the hot plate test and glutamate–induced nociception. The acetic acid–induced writhing method was widely used for the evaluation of peripheral antinociceptive activity. This test was able to determine the antinociceptive effects of compounds or dose levels that might appear inactive in other methods, such as the hot–plate test [13]. Acetic acid induces pain by liberating endogenous substances, as well as some other pain mediators, such as arachidonic acid via cyclooxygenase, and prostaglandin biosynthesis [14]. Because acetic acid indirectly induced the release of endogenous mediators to stimulate the nociceptive neurons that were sensitive to both narcotics analgesia (morphine) and non–steroidal anti–inflammatory drugs such as aspirin [15], it has been established that constriction induced by acetic acid is considered to be a non–selective antinociceptive model. Our results indicated that BHE at the doses of 100, 200 and 800 mg/kg (p.o.) could reduce the number of writhing occurrences in this animal model, showing powerful antinociceptive effects. Interestingly, when the dose of BHE was increased to 800 mg/kg (p.o., n = 10), the inhibition rate was 100%. This indicated that the analgesic intensity interval of BHE was large. The analgesic response at 100 mg/kg was quite similar to that of the standard drug aspirin, and the antinociceptive activity was dose–dependent. However, the results of this writhing test alone do not indicate whether the antinociceptive effect was central or peripheral.

In order to determine this, the tail–flick test was carried out. The tail–flick test is predominantly based on spinal reflex and is considered to be selective for centrally–acting analgesic compounds, while peripherally–acting analgesics are known to be inactive to thermal stimuli [16]. In our investigation, BHE at the doses of 100 and 200 mg/kg (Table 1) showed marked inhibition in thermally–induced hyperalgesia in the tail–flick test. The antinociceptive activity of BHE was measured at different time points, and a time–effect relationship was revealed. The data indicated that the peak antinociceptive activities of BHE and morphine were observed at 60 and 30 minutes post–injection respectively, which is in accordance with reference previous report [17]. In addition, because the Automatic Analgesia Tail–Flick apparatus (UGO BASILE S.R.L., Model: 7360, Italy) was used, measurement errors were diminished.

To further confirm the role of the central nervous system in the antinociceptive activity of BHE, the hot plate test was employed. The hot plate test is generally used for centrally acting analgesic drugs, such as morphine, while peripherally acting analgesics are ineffective in this test [18]. In our research, BHE at the doses of 100 and 200 mg/kg (Table 2) significantly prolonged the mice’s duration on the hot plate. Morphine (10 mg/kg, s.c.) was used as a positive control drug and demonstrated a stronger analgesic effect than BHE. Therefore, the effect of BHE on the response to the hot–plate provided a further confirmation of its central effect.

For the assessment of the involvement of the opioid system in the analgesic activity of BHE, a non–selective opioid receptor antagonist, naltrexone (5 mg/kg, s.c.) was administered. The opioid system is involved in many central nervous system functions and has long been known to play a central role in regulating the experiences of physical pain. Endogenous opioid neurotransmission increases during physical pain and lessens the affective experience of pain [19]. The data obtained in the hot plate test showed that naltrexone was not able to reverse the antinociceptive effect produced by BHE. Because the antinociceptive mechanism of BHE did not work through the opioid receptor, BHE may be a good candidate for future clinical trials. It is well known that although morphine is a highly potent analgesic, there is a fatal side effect (i.e., drug addiction) for its long–term use. Therefore there is a need for development of new non–opioid analgesics and our results suggest that BHE is a potential candidate for this purpose.

The oral administration of BHE also produced a dose–dependent inhibition of the nociceptive response induced by glutamate injection into the hind paw of mice. The glutamate–induced nociception response involves peripheral, spinal and supraspinal sites of action with glutamate receptors, such as AMPA (α–amino–3–hydroxy–5–methyl–4–isoxazole–propionic acid), kainate and NMDA (N–methyl–D–aspartic acid) receptors. This interaction contributes to modulating this nociceptive response [20], so the antinociceptive activity caused by BHE may be associated with the glutamatergic system. The current study also investigated the involvement of the cGMP pathway in the antinociceptive activity of BHE. Physiological functions, such as pain and analgesia, are influenced by the cellular level of cGMP, which is regulated by the action of sGC (soluble guanylyl cyclase), which is mediated by nitric oxide (NO) [21]. To evaluate the involvement of cGMP in the analgesic activity of BHE, methylene blue (MB), a guanylyl cyclase inhibitor, was administered prior to inducing nociception with an intraperitoneal injection of acetic acid. The results demonstrated that methylene blue significantly reduced the nociception caused by acetic acid and also enhanced the antinociceptive effect of BHE. It has been suggested that methylene blue promotes antinociceptive activity by inhibiting peripheral NO and sGC that resulted from the NO interference [21]. When methylene blue and BHE were used together, the antinociceptive effect was increased in the acetic acid–induced writhing model compared with the group that received BHE only. This strongly indicates that the antinociceptive effect of BHE involves the cGMP pathway. The results also indicated that the antinociceptive effect of BHE may involve the participation of the ATP–sensitive K^+^ channel pathway because glibenclamide, an ATP–sensitive K^+^ channel antagonist, could partially reverse the antinociceptive activity of BHE. The antinociceptive action of BHE might relate to ATP–sensitive K^+^ channel opening and the subsequent efflux of K^+^ ions and membrane repolarization and/or hyperpolarization [22].

In this study, one phenolic acid (gallic acid), one lignin (peperomin B) and five flavonoids (quercetin, fisitin, luteolin, farrerol and racemate) were isolated from BHE. These compounds were characterized by ^1^H NMR and MS spectral data. According to the literature obtained by searching PubMed, Google Scholar, Web of Science and Science Direct, gallic acid and peperomin B have not demonstrated analgesic effects. Therefore, the antinociceptive activity of BHE is most likely due to the presence of the flavonoids. Flavonoids may increase the amount of endogenous serotonin or interact with 5–HT_2_ and 5–HT_3_ receptors, which may be involved in the mechanism of central analgesic activity [23]. There are also reports that flavonoids may work by targeting prostaglandins in analgesic activity [24]. BHE showed significant analgesic activity in this experimental model throughout the study, which may be due to its high flavonoid content.

## Conclusion

This study demonstrated that the ethanol extract of the aerial parts of *B. glauca* subsp. *hupehana* possessed significant antinociceptive activity in both chemically–induced (abdominal constriction induced by acetic acid; glutamate–induced nociception) and heat–induced (tail–flick test; hot plate test) pain models. The antinociceptive mechanism may involve the cGMP pathway and the ATP–sensitive K^+^ channel pathway. The central antinociceptive effect was not antagonized by naltrexone. The antinociceptive activity of BHE was most likely due to the presence of flavonoids. Further studies are needed to fully understand the mechanisms of action of BHE and to find the bioactive compound that may act as a lead for drug development.

## Supporting Information

S1 Fig Chemical structure and ^1^H NMR spectral data of gallic acid. (TIF)

S2 Fig MS spectral data of gallic acid. (TIF)

S3 Fig Chemical structure and ^1^H NMR spectral data of peperomin B. (TIF)

S4 Fig MS spectral data of peperomin B. (TIF)

S5 Fig Chemical structure and ^1^H NMR spectral data of quercetin. (TIF)

S6 Fig MS spectral data of quercetin. (TIF)

S7 Fig Chemical structure and ^1^H NMR spectral data of fisetin. (TIF)

S8 Fig MS spectral data of fisetin. (TIF)

S9 Fig Chemical structure and ^1^H NMR spectral data of luteolin. (TIF)

S10 Fig MS spectral data of luteolin. (TIF)

S11 Fig Chemical structure and ^1^H NMR spectral data of farrerol. (TIF)

S12 Fig MS spectral data of farrerol. (TIF)

S13 Fig Chemical structure and ^1^H NMR spectral data of garbanzol. (TIF)

S14 Fig MS spectral data of garbanzol. (TIF)
