# Supplementary figures and images for: Phytochemical Composition and Antinociceptive Activity of Bauhinia glauca subsp. hupehana in Rats
Source: PLoS One. 2015 Feb 6;10(2):e0117801. doi: 10.1371/journal.pone.0117801 (PMC4320050; doi:10.1371/journal.pone.0117801)

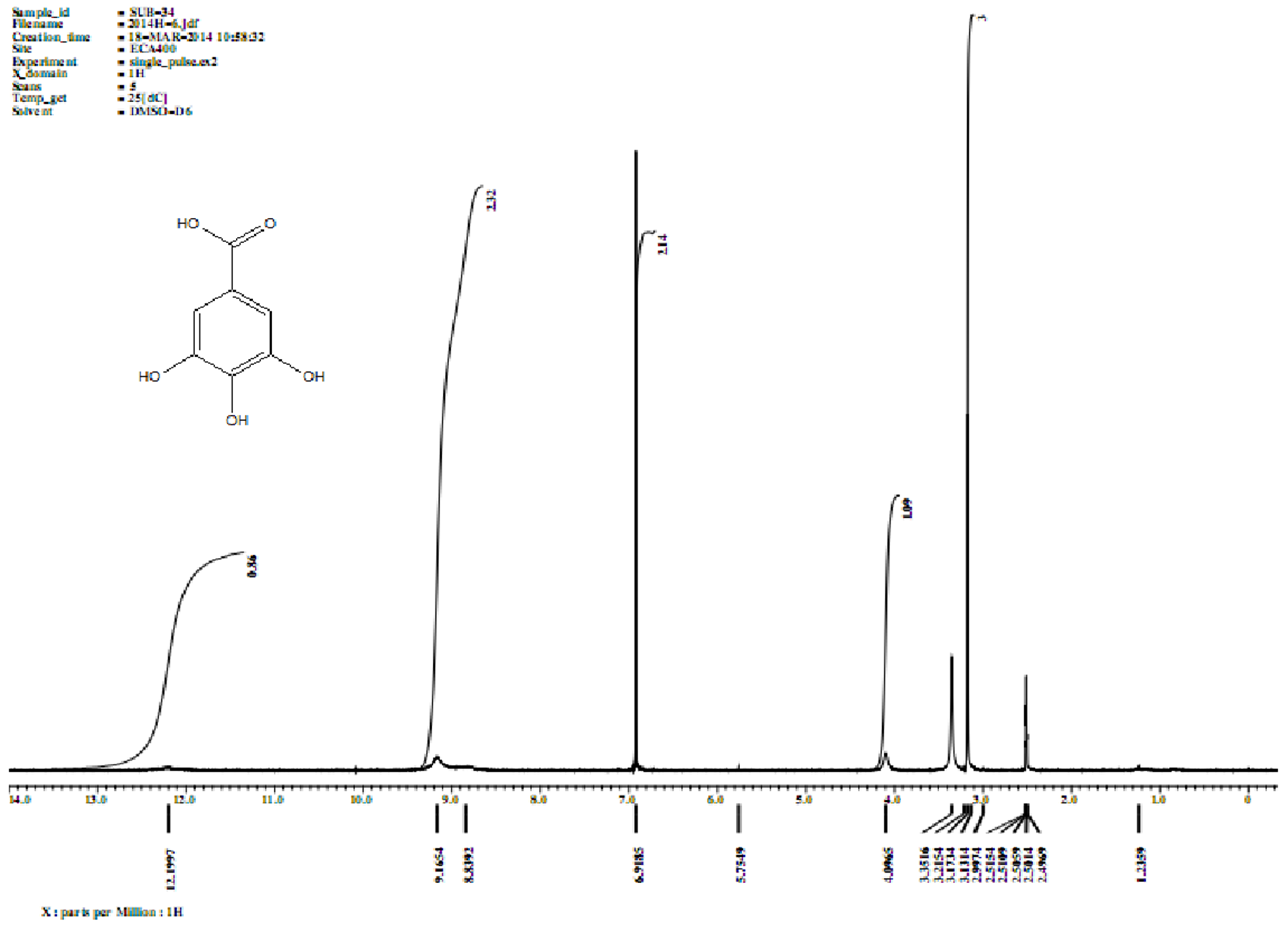

Supplement: S1 Fig — (TIF) [file pone.0117801.s001.tif]

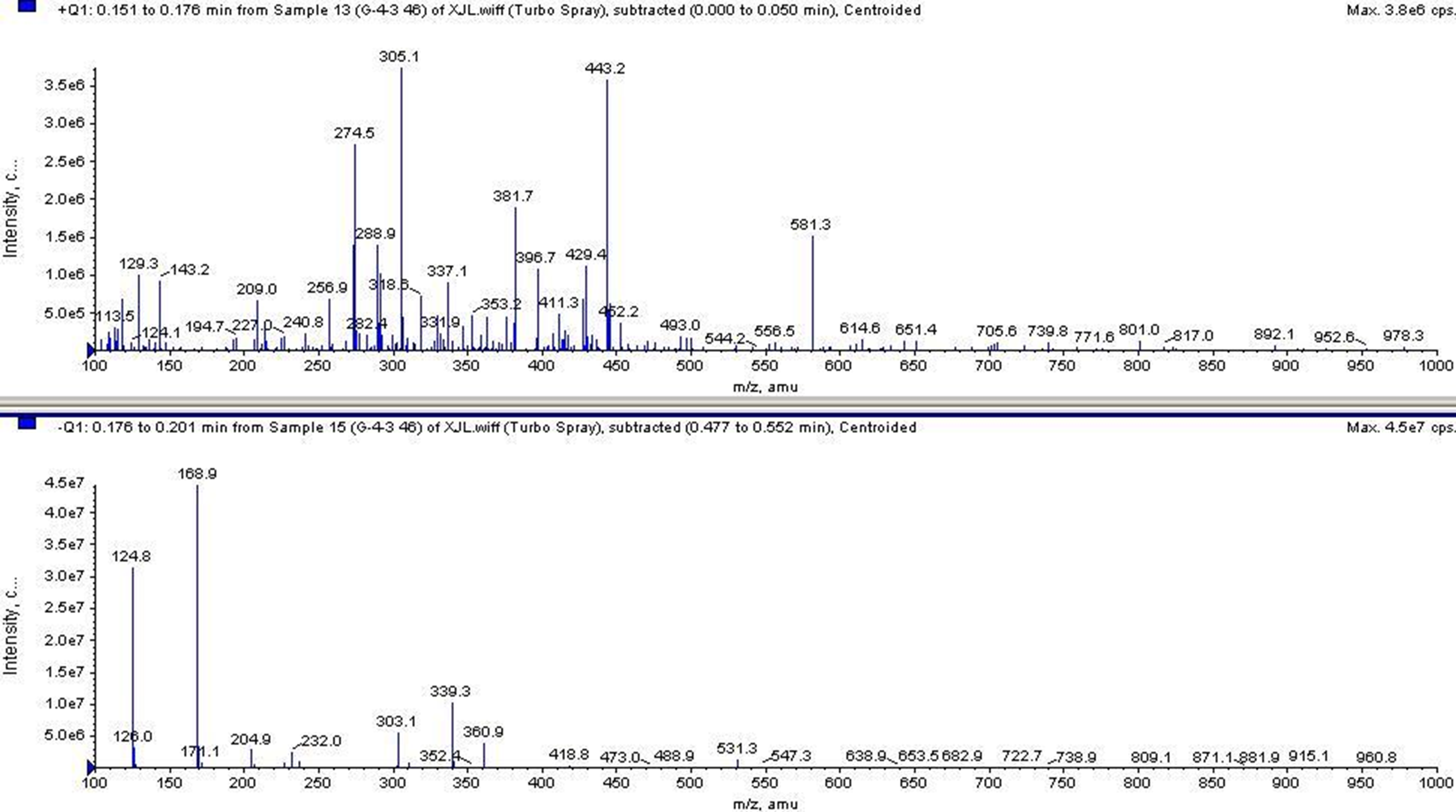

Supplement: S2 Fig — (TIF) [file pone.0117801.s002.tif]

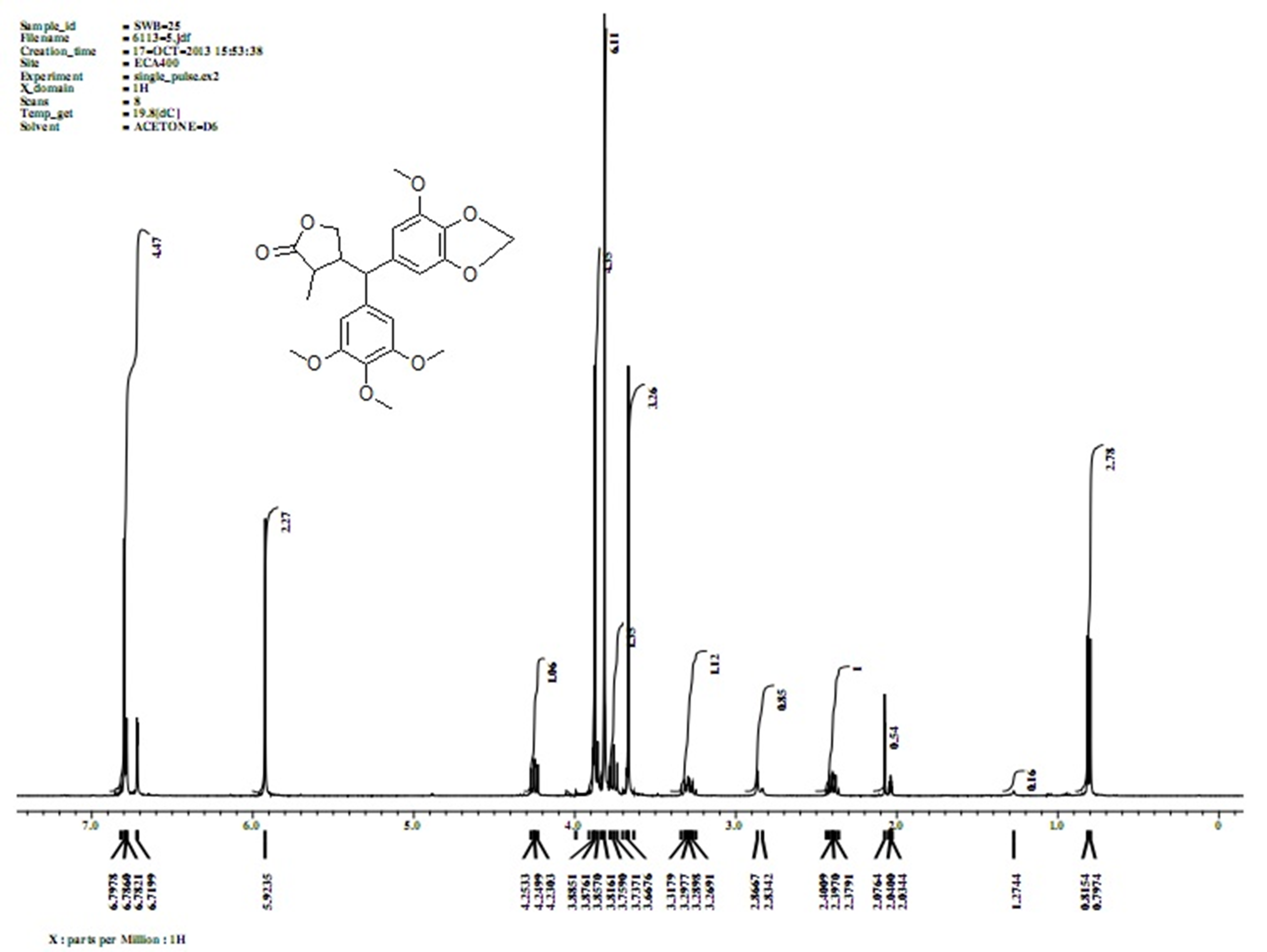

Supplement: S3 Fig — (TIF) [file pone.0117801.s003.tif]

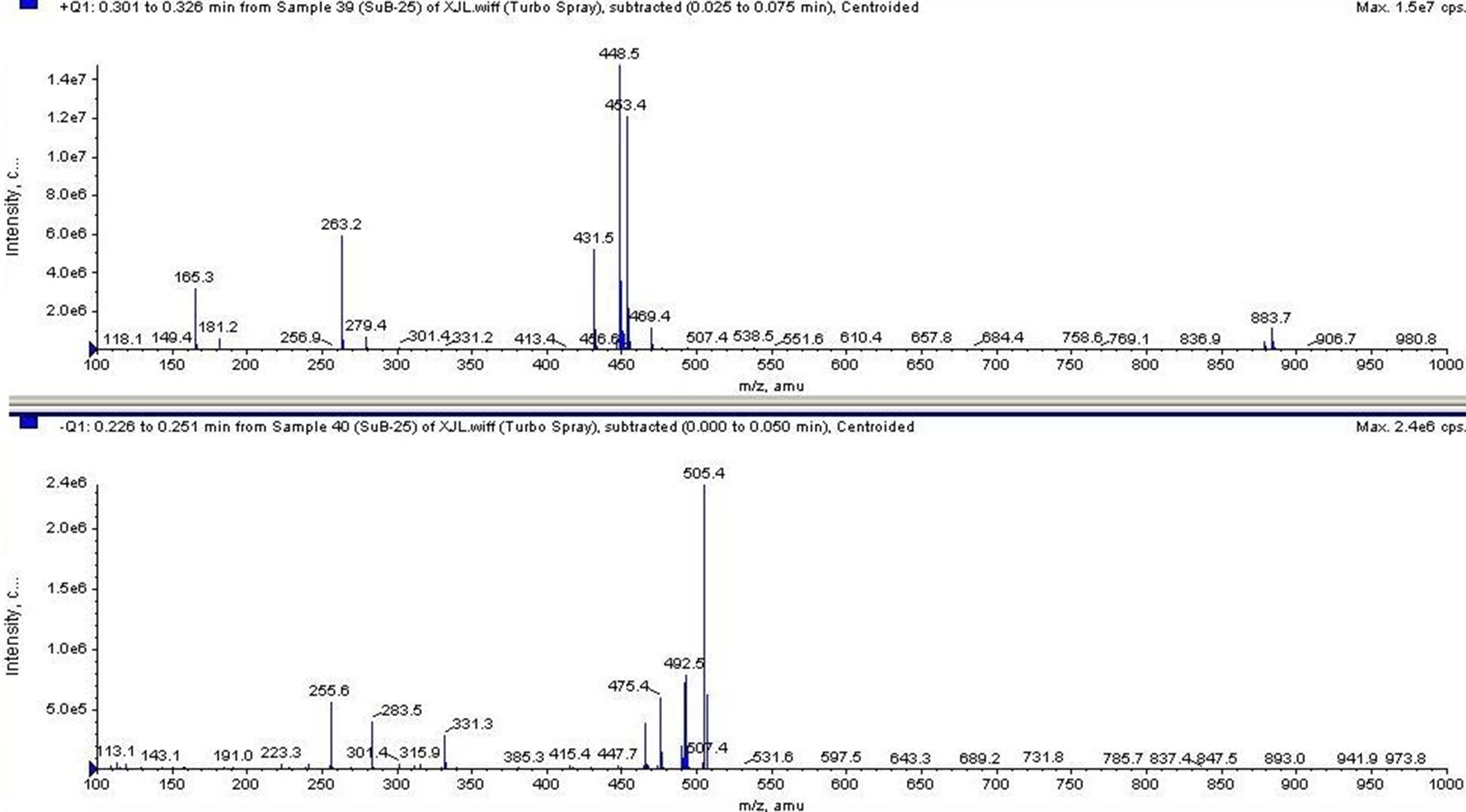

Supplement: S4 Fig — (TIF) [file pone.0117801.s004.tif]

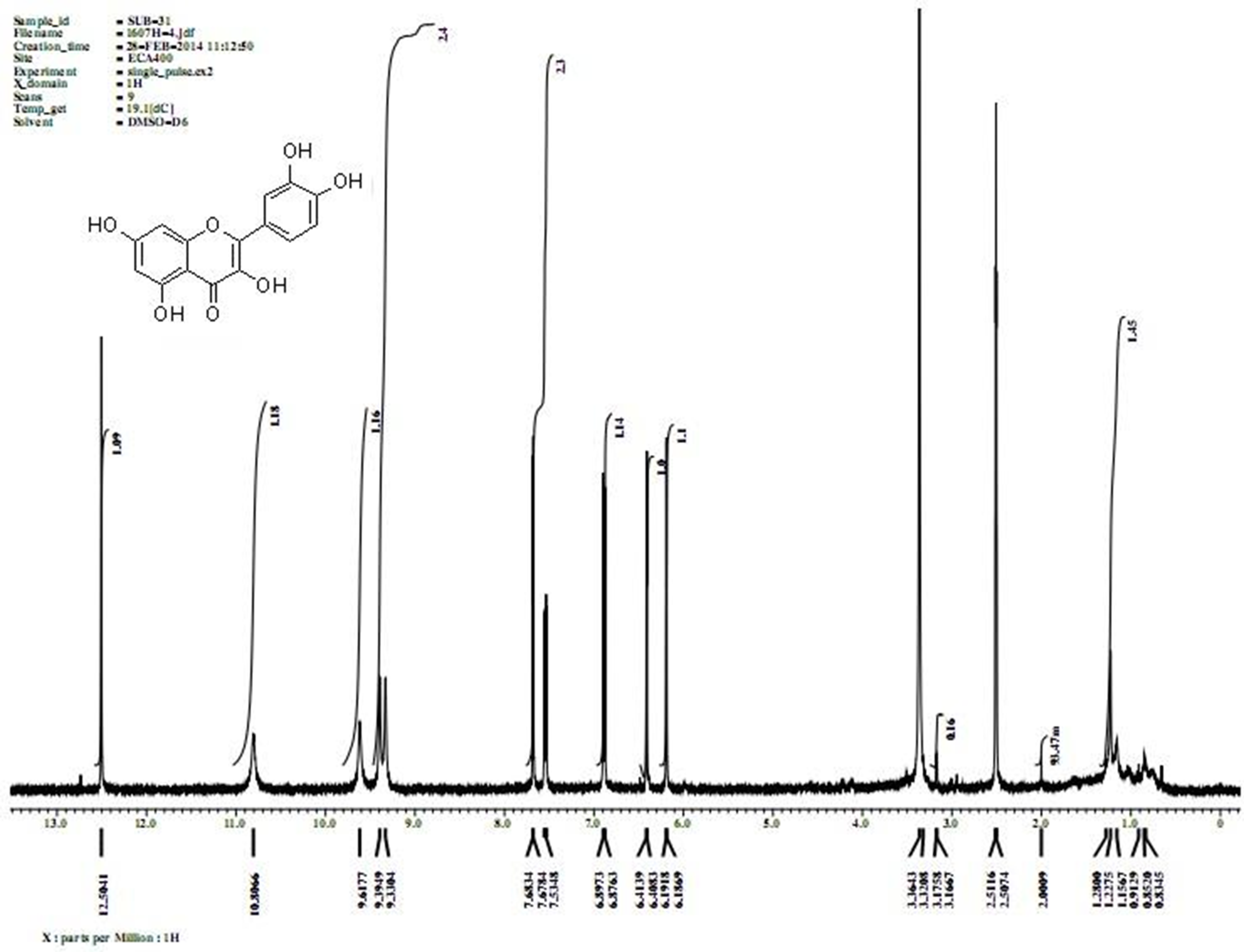

Supplement: S5 Fig — (TIF) [file pone.0117801.s005.tif]

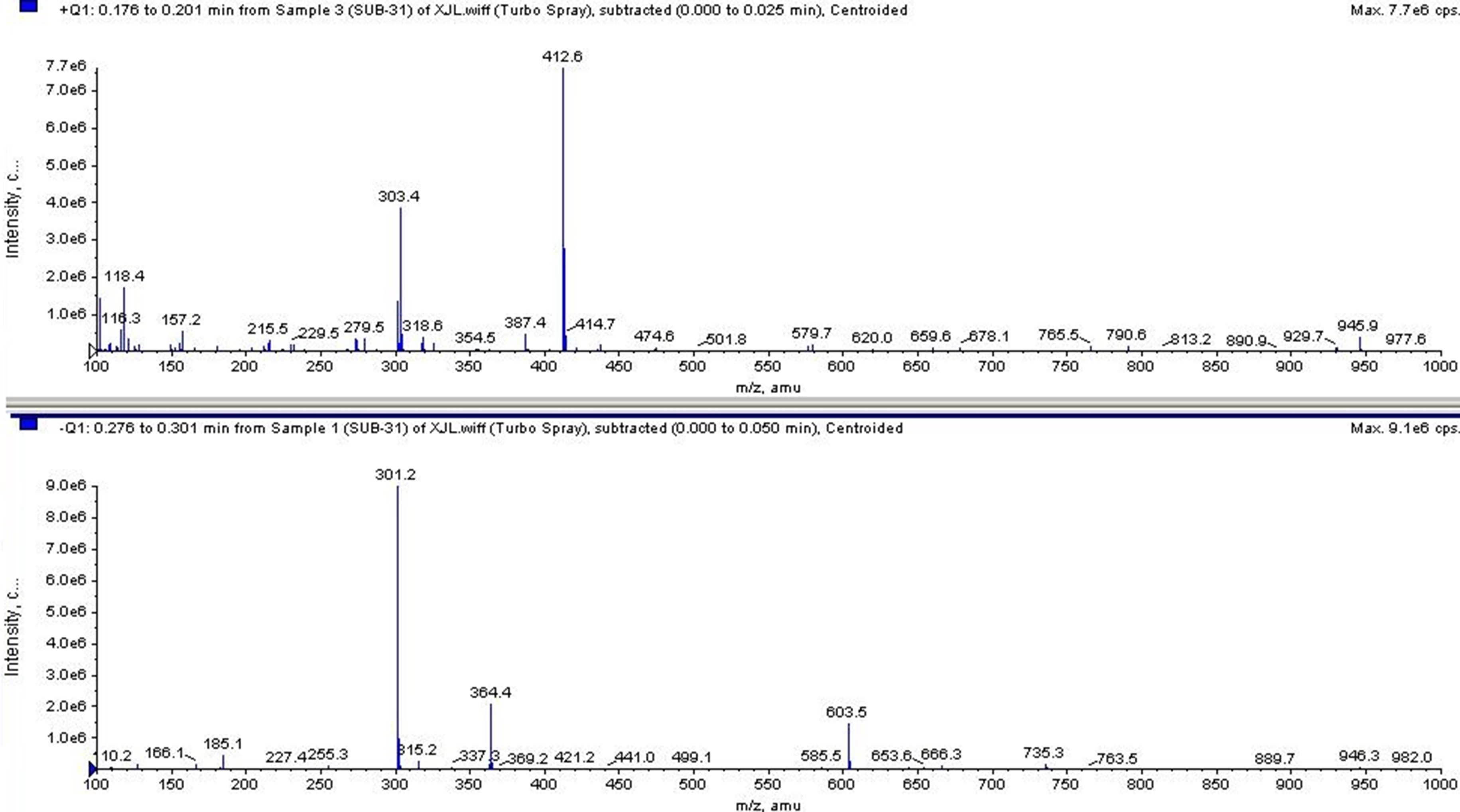

Supplement: S6 Fig — (TIF) [file pone.0117801.s006.tif]

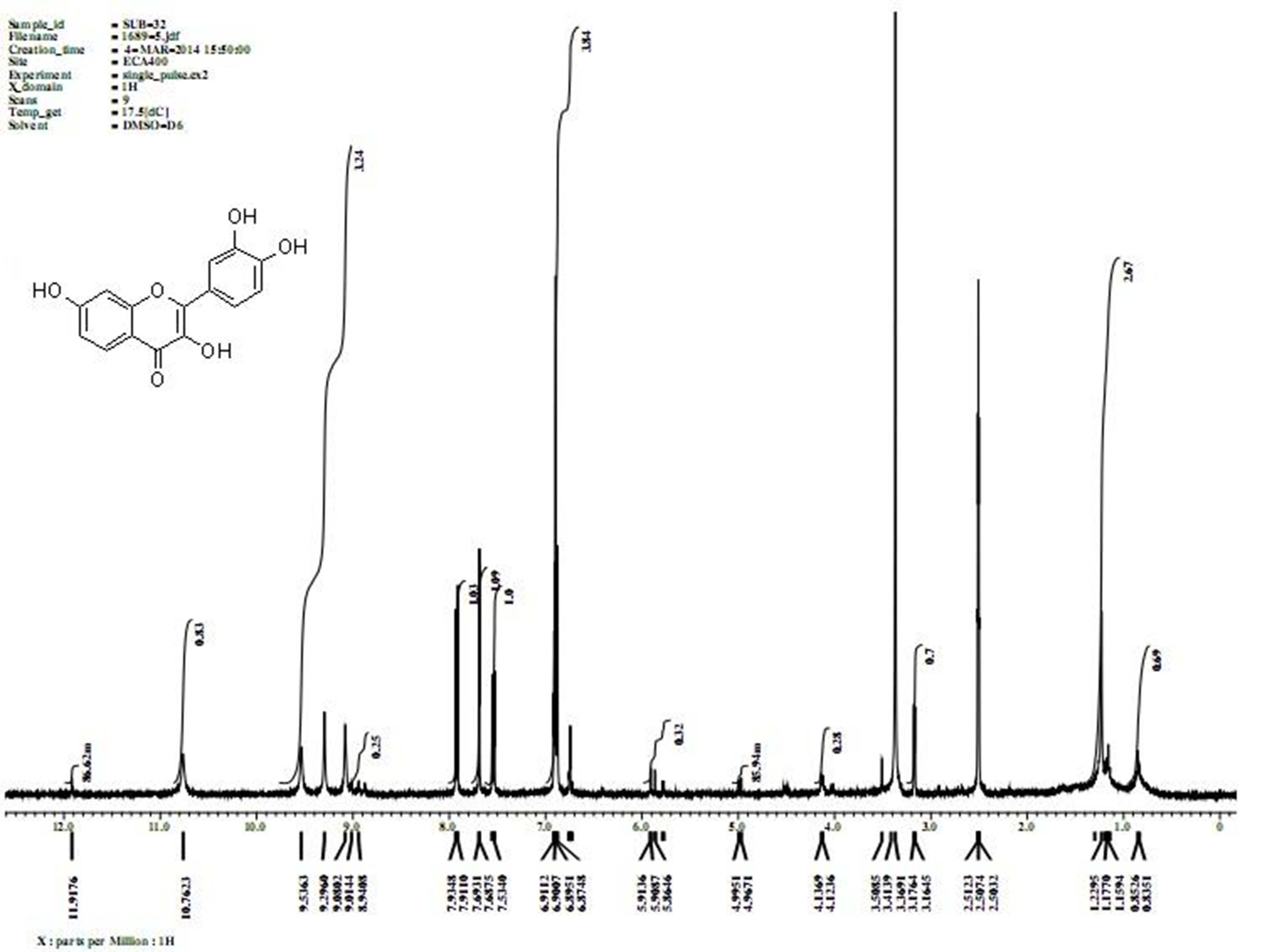

Supplement: S7 Fig — (TIF) [file pone.0117801.s007.tif]

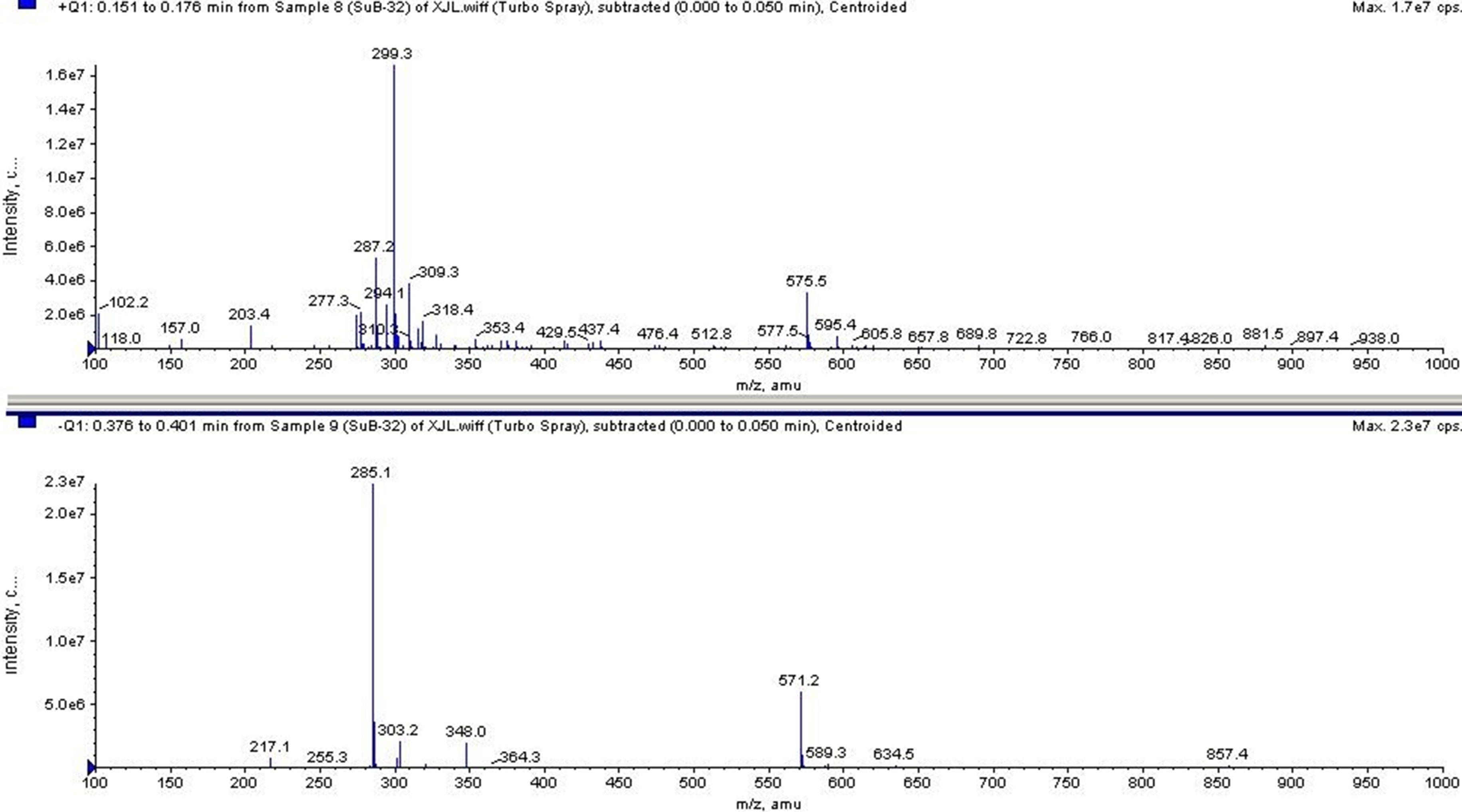

Supplement: S8 Fig — (TIF) [file pone.0117801.s008.tif]

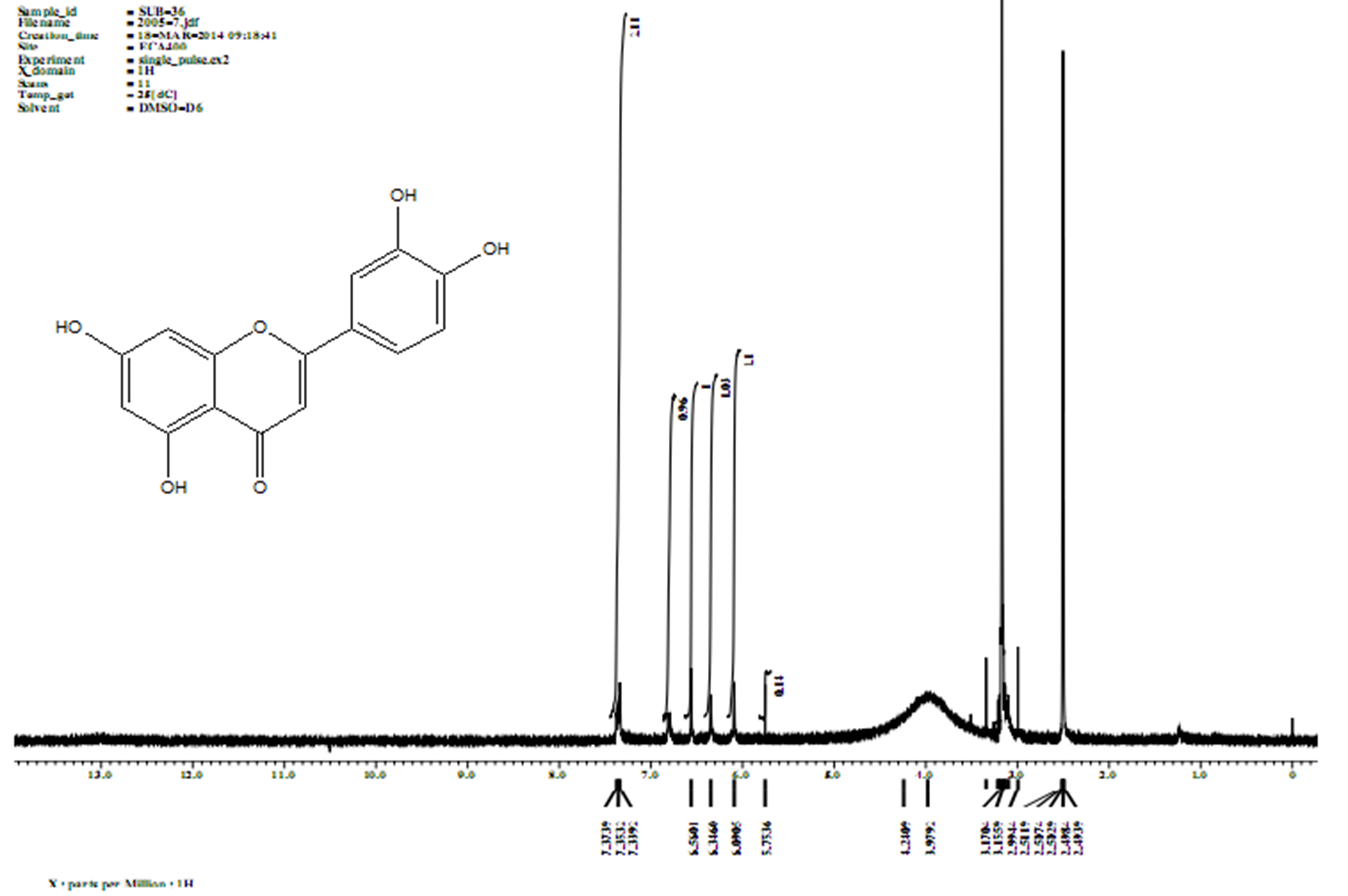

Supplement: S9 Fig — (TIF) [file pone.0117801.s009.tif]

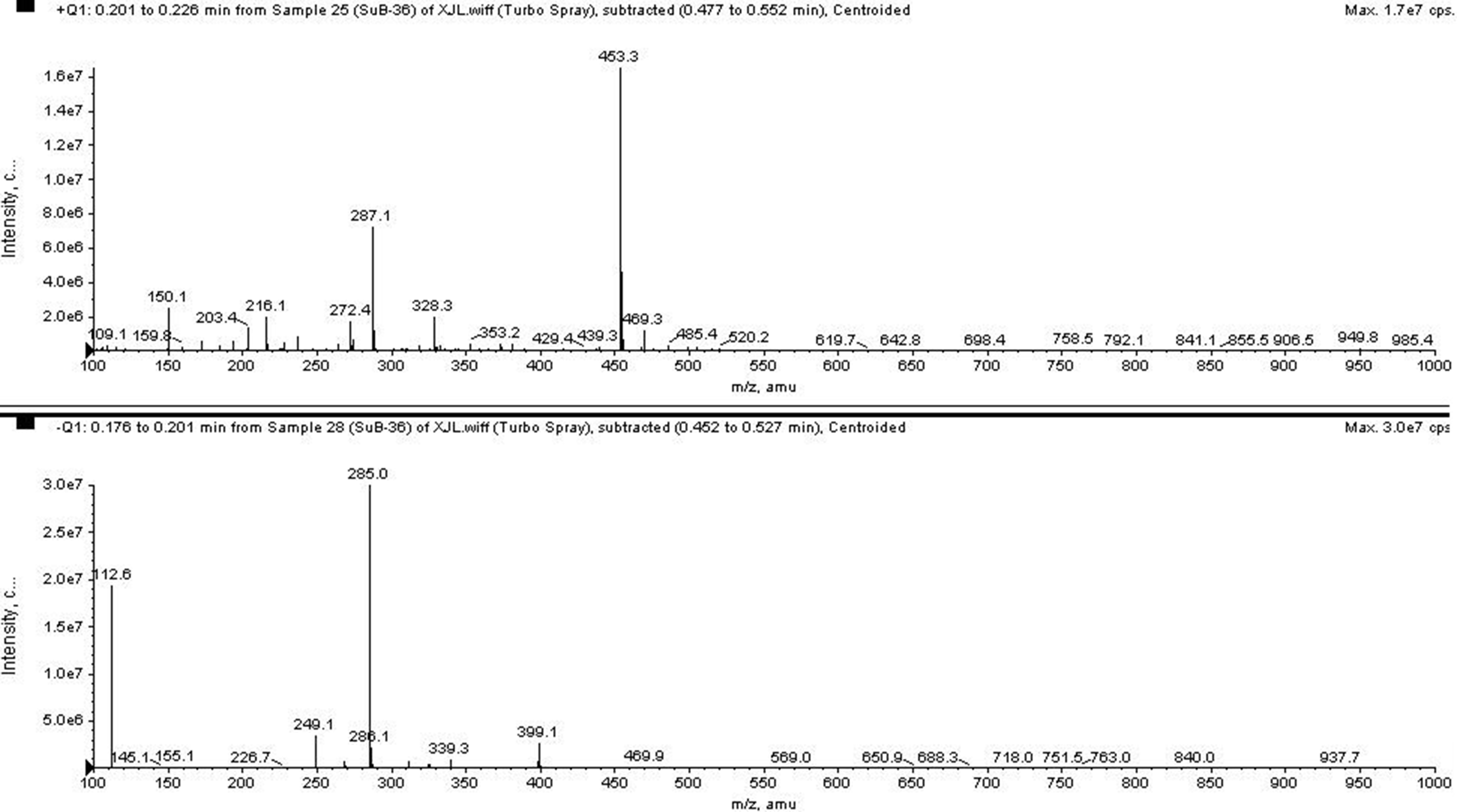

Supplement: S10 Fig — (TIF) [file pone.0117801.s010.tif]

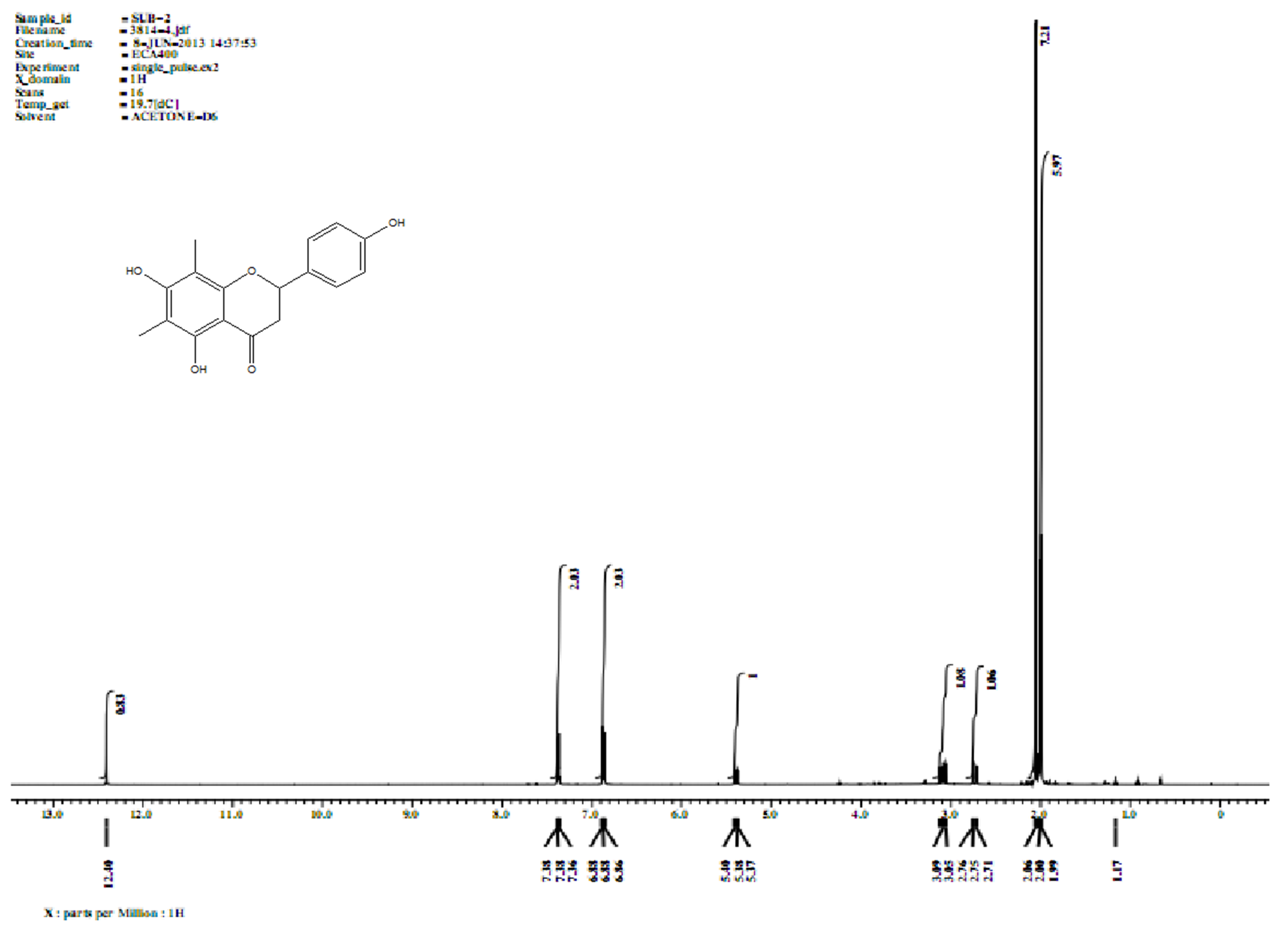

Supplement: S11 Fig — (TIF) [file pone.0117801.s011.tif]

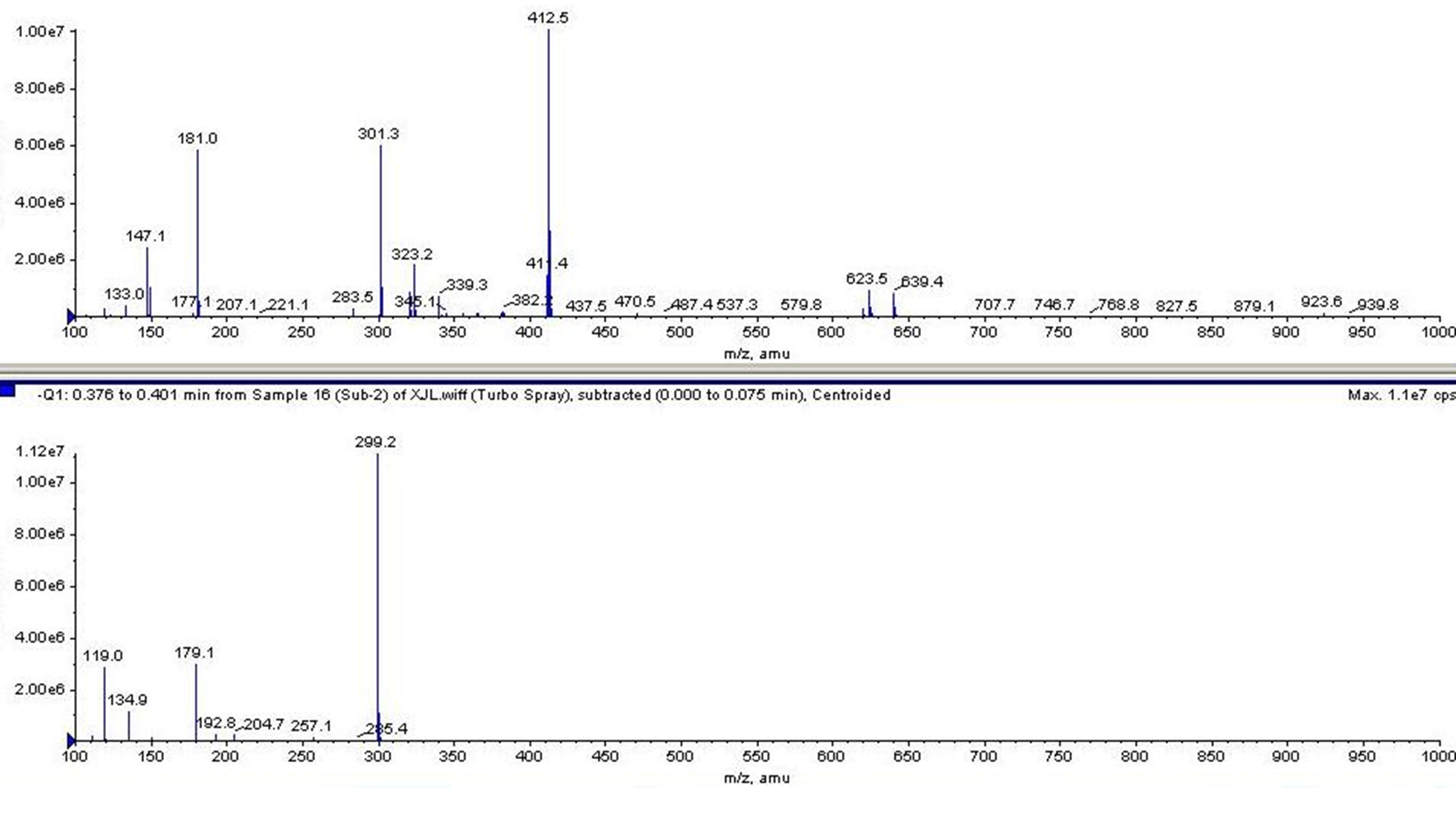

Supplement: S12 Fig — (TIF) [file pone.0117801.s012.tif]

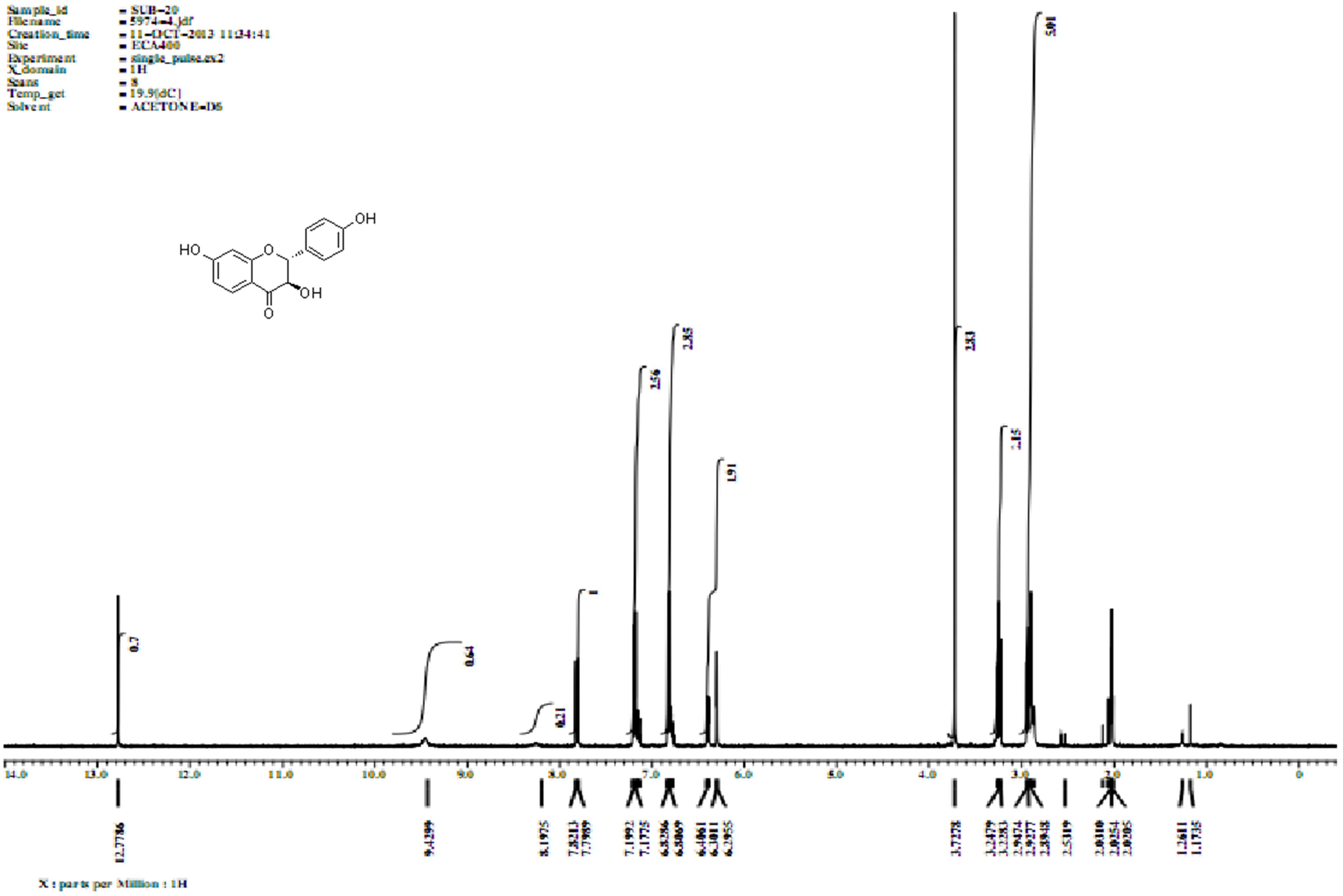

Supplement: S13 Fig — (TIF) [file pone.0117801.s013.tif]

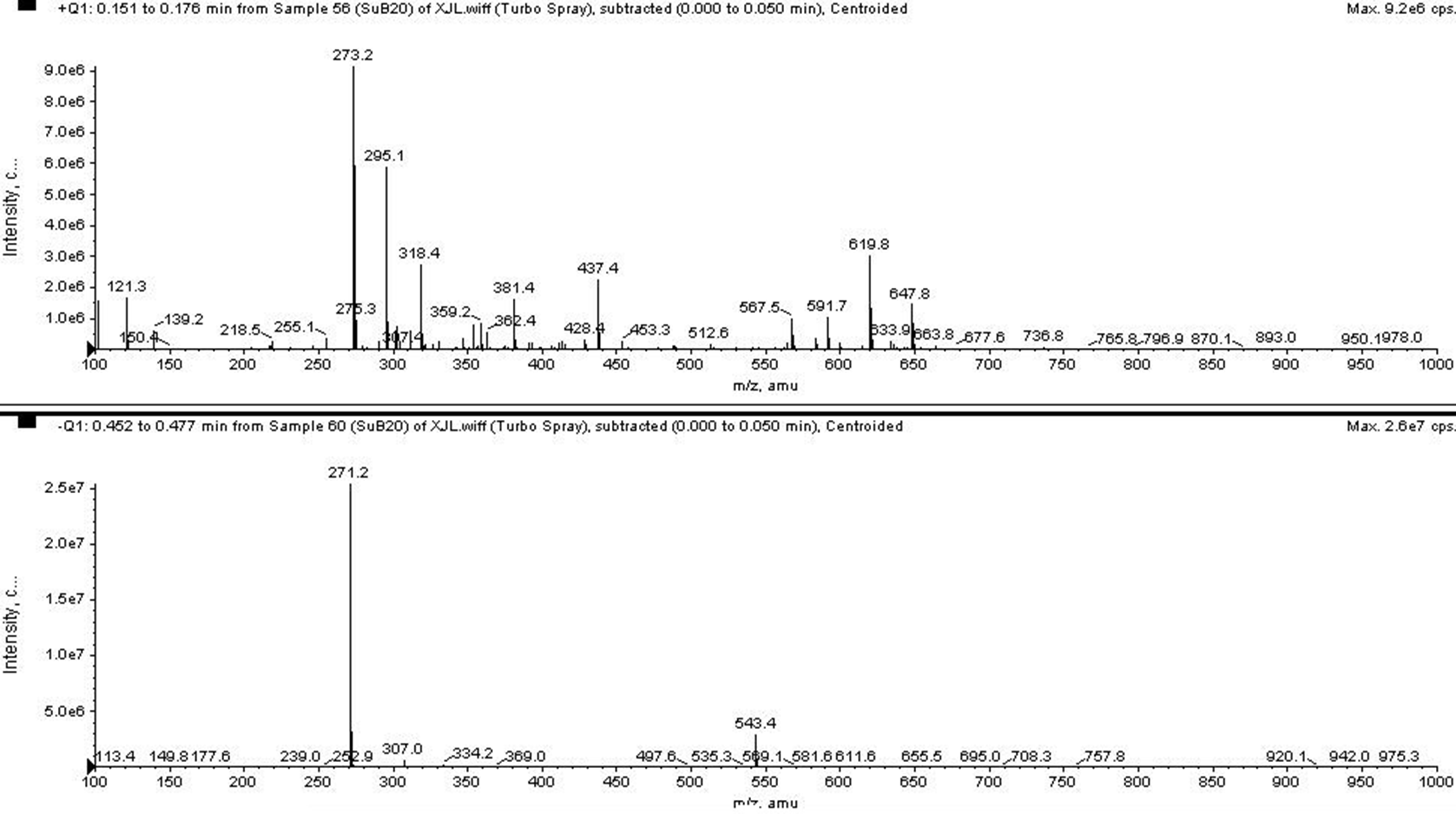

Supplement: S14 Fig — (TIF) [file pone.0117801.s014.tif]
